# Iron affects Ire1 clustering propensity and the amplitude of endoplasmic reticulum stress signaling

**DOI:** 10.1242/jcs.201715

**Published:** 2017-10-01

**Authors:** Nir Cohen, Michal Breker, Anush Bakunts, Kristina Pesek, Ainara Chas, Josepmaria Argemí, Andrea Orsi, Lihi Gal, Silvia Chuartzman, Yoav Wigelman, Felix Jonas, Peter Walter, Robert Ernst, Tomás Aragón, Eelco van Anken, Maya Schuldiner

**Affiliations:** 1 Department of Molecular Genetics, Weizmann Institute of Science, Rehovot 7610001, Israel; 2 The Rockefeller University, New York, NY 10065, USA; 3 Division of Genetics and Cell Biology, San Raffaele Scientific Institute, Ospedale San Raffaele, Via Olgettina 58, 20132, Milan, Italy; 4 Institute of Biochemistry and Buchmann Institute for Molecular Life Sciences, Goethe University Frankfurt, Max-von-Laue Str. 15, 60438 Frankfurt, Germany; 5 Center for Applied Medical Research, Department of Gene Therapy and Regulation of Gene Expression. University of Navarra, 55 Pio XII St. 31008 Pamplona, Spain; 6 Department of Biochemistry & Biophysics, University of California San Francisco and Howard Hughes Medical Institute, San Francisco, CA 94143, USA; 7 Center for Molecular Signaling, Institute of Medical Biochemistry and Molecular Biology, Saarland University, 66421 Homburg, Germany

**Keywords:** Ire1, Iron, Heme, Sterol, *Saccharomyces cerevisiae*, UPR

## Abstract

The unfolded protein response (UPR) allows cells to adjust secretory pathway capacity according to need. Ire1, the endoplasmic reticulum (ER) stress sensor and central activator of the UPR is conserved from the budding yeast *Saccharomyces cerevisiae* to humans. Under ER stress conditions, Ire1 clusters into foci that enable optimal UPR activation. To discover factors that affect Ire1 clustering, we performed a high-content screen using a whole-genome yeast mutant library expressing Ire1–mCherry. We imaged the strains following UPR induction and found 154 strains that displayed alterations in Ire1 clustering. The hits were enriched for iron and heme effectors and binding proteins. By performing pharmacological depletion and repletion, we confirmed that iron (Fe^3+^) affects UPR activation in both yeast and human cells. We suggest that Ire1 clustering propensity depends on membrane composition, which is governed by heme-dependent biosynthesis of sterols. Our findings highlight the diverse cellular functions that feed into the UPR and emphasize the cross-talk between organelles required to concertedly maintain homeostasis.

## Abstract (precis)

**Highlighted Article:** To respond to folding stress in the ER, cells activate the conserved sensor Ire1. We show that iron is required for optimal Ire1 activation and suggest this is because iron is required for ergosterol biosynthesis.

## INTRODUCTION

The unfolded protein response (UPR) is a homeostatic response pathway that adjusts the endoplasmic reticulum (ER) protein-folding capacity according to need. The UPR is common to all eukaryotes despite variance in sensing machineries and regulation (Walter and Ron, 2011). The UPR signaling mechanism that is initiated by Ire1 is present from *Saccharomyces cerevisiae* (from here on referred to simply as ‘yeast’) to humans (IRE1α, encoded by the *ERN1* gene) (Cox et al., 1993; Mori et al., 1993; Tirasophon et al., 1998; Walter and Ron, 2011). Ire1 is a single-pass ER membrane protein that senses stress such as the accumulation of misfolded proteins (Cox et al., 1997) or alterations in membrane composition (Halbleib et al., 2017; Pineau et al., 2009; Promlek et al., 2011; Surma et al., 2013). Upon activation, Ire1 dimerizes to activate its kinase domain in the cytosol and trans autophosphorylates (Shamu and Walter, 1996; Tirasophon et al., 1998). Phosphorylation activates the endonuclease domain of Ire1 driving a non-conventional splicing reaction, which removes a single intron from *HAC1* mRNA in yeast (Cox and Walter, 1996; Yoshida et al., 1998) and its orthologous *XBP1* mRNA in mammals (Yoshida et al., 2001). The splicing reaction enables the creation of the mature proteins, which act as transcription factors for gene promoters with a UPR element (UPRE) in yeast (Cox and Walter, 1996) or an ER stress element (ERSE) in mammals (Yoshida et al., 1998).

Over the past years, it has become evident that both Ire1 and Hac1/Xbp1 are regulated on multiple levels (Gardner et al., 2013; Pineau et al., 2009). One regulatory aspect of Ire1 function is its propensity to cluster into foci upon ER stress in both yeast (Aragón et al., 2009; Kimata et al., 2007; van Anken et al., 2014) and mammals (Li et al., 2010). While this clustering is not essential for UPR initiation, it has a role in enabling optimal activation (Li et al., 2010) by providing a platform to which the *HAC1* mRNA can be targeted (Aragón et al., 2009) and docked (van Anken et al., 2014), ensuring efficiency and specificity of the splicing reaction (van Anken et al., 2014).

We decided to exploit formation of Ire1 clusters as a striking visual output for the extent of UPR activation, and set out to perform a high-content screen using a library of yeast strains in which single genes were ablated or had reduced function. We found that the loss of many genes affected the dynamics of Ire1 clustering. Surprisingly, the hits were highly enriched in genes associated with iron and heme metabolism. In line with the genetic evidence, we showed that iron (Fe^3+^) availability strongly influenced whether a productive UPR could be mounted in both yeast and human cells. We continued to show that heme and ergosterol biosynthesis enzymes are required for optimal clustering. We thus raise the hypothesis that iron levels affect heme biosynthesis that, in turn, determines membrane composition (since heme is a co-factor of enzymes in sterol biosynthesis as well as enzymes that affect lipid saturation), and that membrane composition affects Ire1 clustering. Our findings support previous observations on the role of membrane composition in UPR activation (Volmer and Ron, 2015) as well as our recent observations on how Ire1 senses bilayer stress (Halbleib et al., 2017). Thus, iron and heme levels, next to ATP levels (McClellan et al., 1998; Todd-Corlett et al., 2007), the level of unfolded proteins in the ER lumen (Gardner and Walter, 2011; McClellan et al., 1998; Todd-Corlett et al., 2007) and the extent of BiP (also known as GRP78, and encoded by the *HSPA* gene; Kar2 in yeast) binding to Ire1 (Okamura et al., 2000; Todd-Corlett et al., 2007) are all determinants of Ire1 activation and thereby can play a role in the fine-tuning of the UPR.

## RESULTS

### A high-content screen uncovers multiple effectors of Ire1 clustering

**Fig. 1.**
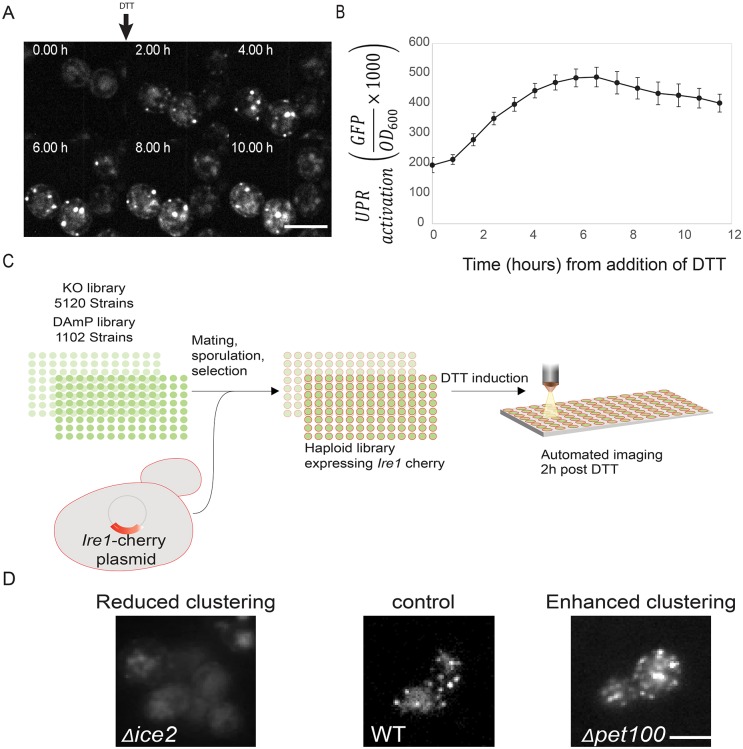
**A systematic high-content screen reveals effectors of Ire1 clustering.** (A) Ire1–mCherry was visualized over time following induction of the UPR by 2 mM DTT and shows clustering as expected. (B) UPR activity was measured over time using a UPRE-GFP reporter following induction of UPR by 2 mM DTT. UPR activity increases until an activation plateau is reached. Results are mean±s.d. (*n*=9). (C) Work flow for systematic screen to uncover Ire1–mCherry clustering effectors. (D) Hits from the Ire1–mCherry screen were divided into two phenotypic groups. WT, wild type. Scale bars: 5 μm.

To monitor the dynamics of UPR activation through visualization of Ire1 clustering, we created a yeast strain expressing Ire1 that is tagged with mCherry in the cytosolic linker that tethers the kinase domain of Ire1 to the transmembrane region. This unique tag localization preserves all Ire1 functions (Aragón et al., 2009). We confirmed that Ire1–mCherry, when exposed to the reducing agent dithiothreitol (DTT) that induces ER stress, redistributed from its diffuse localization throughout the ER membrane into small discrete punctate structures that were easy to visualize after 2 h. The Ire1–mCherry foci then grew in size, and Ire1–mCherry remained clustered for the entire duration of the experiment, spanning over more than 10 h (Fig. 1A).

To measure how such localization dynamics relate to downstream UPR activation, we also employed a strain that harbors the UPRE-GFP reporter cassette (Cox and Walter, 1996). In this strain, GFP fluorescence can be used as a proxy for the extent of UPR activation. Indeed, by 2 h post-DTT addition, we could already observe an increase in the induction that continued until 6 h after DTT addition, when GFP levels reached a plateau (Fig. 1B). It is important to mention that UPR activation probably starts before clustering can be observed and that there is a lag time between Ire1 sensing and the increase in UPRE-regulated GFP due to the time it takes to transcribe, translate and fold the GFP. Hence, Ire1–mCherry clustering and UPRE-GFP increase each have their spatio-temporal restrictions. However, as both assays identified 2 h as the earliest time point in which a clear UPR activation by these reporters could be assayed, we chose this time point for our screen.

To identify proteins that affect Ire1 clustering dynamics, we used a library of deletions in all non-essential yeast genes (Giaever et al., 2002) and a library of hypomorphic alleles of all essential ones (Breslow et al., 2008). Using a method for automated mating, sporulation and selection of desired haploids, we tailored the library such that each strain expressed Ire1–mCherry (Cohen and Schuldiner, 2011; Tong et al., 2001) (Fig. 1C). After exposure of the strains to DTT for 2 h, we imaged the entire collection using a high-content imaging platform (Breker et al., 2013) and manually inspected and curated all of the images taking into consideration the number, the intensity and the size of the clusters. We identified 154 strains in which Ire1–mCherry clustering was altered as compared to a control strain (for a full list of hits and their phenotypes see Table S1). We organized the hits into two groups (Fig. 1D): ‘enhanced clustering’ or ‘reduced clustering’. The large number of hits exemplifies the complexity of regulating a homeostatic response. However, it also poses an experimental difficulty in differentiating direct from indirect effects.

### Genes involved in iron homeostasis affect Ire1 localization

**Fig. 2.**
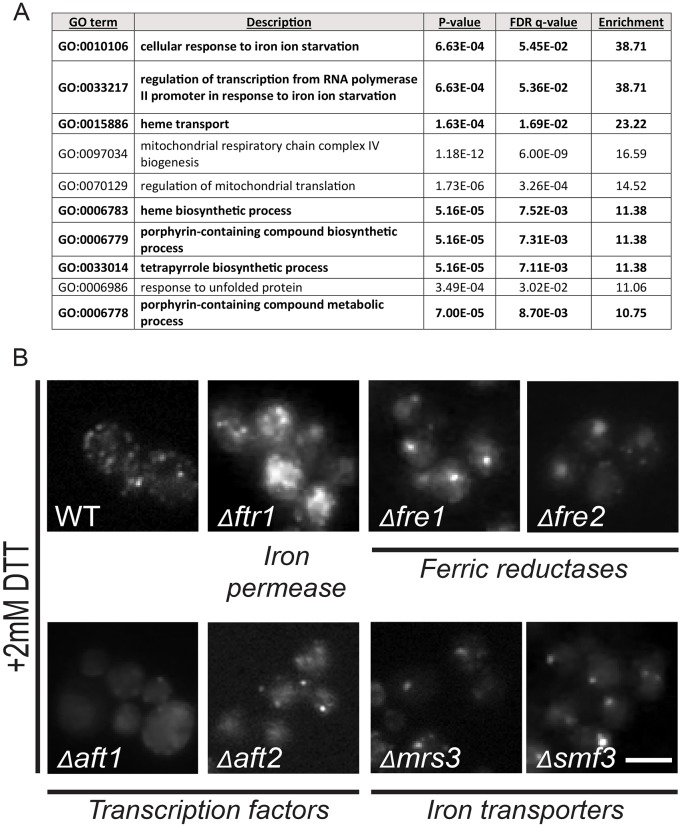
**Iron homeostasis affects Ire1–mCherry clustering.** (A) GO term enrichment of the hits in the screen based on the GOrilla analysis website. The table shows the ten highest enriched categories excluding redundant GO terms. In bold are all iron- and heme-related processes. (B) Deletion of several iron homeostasis genes disrupts Ire1–mCherry clustering following ER stress (2 mM DTT) (images from original screen). WT, wild type. Scale bar: 5 μm.

To assess whether an entire cellular process was over-represented in our data, we performed a GO term enrichment analysis. The analysis uncovered a strong enrichment for processes that are related to iron and heme (with heme homeostasis itself being a major iron-dependent process) (Fig. 2A).

Indeed, there were multiple effectors of iron levels, distribution and homeostasis among the hits from our screen. For example, deletions of the iron transporters Mrs3 and Smf3 caused a dramatic reduction in Ire1–mCherry clustering (Fig. 2B). Similarly, deletions in other genes that govern iron homeostasis, such as the iron permease Ftr1, the ferric reductases Fre1 and Fre2 and the iron-regulon transcription factors Aft1 and Aft2, all caused a decreased clustering phenotype (Fig. 2B). The dependence on iron homeostasis to mount a full-blown UPR has not been previously identified, hence we embarked on verifying the suggested connection.

### Iron levels affect dynamics of Ire1 clustering and UPR activation

**Fig. 3.**
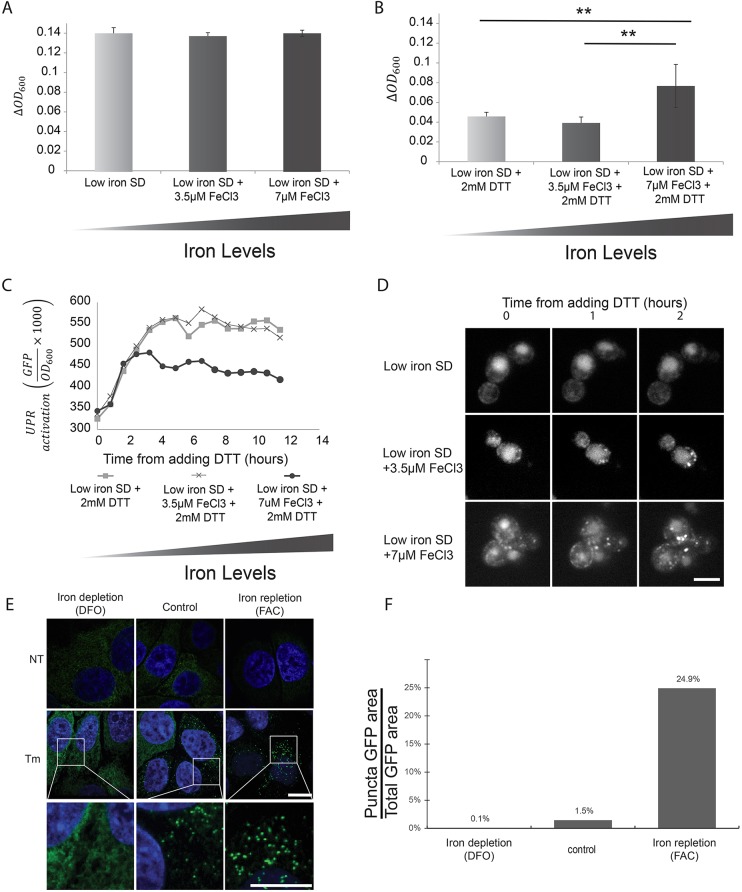
**Iron is essential for mounting a productive UPR.** (A) Iron depletion or repletion does not affect yeast growth under non-ER stress conditions as assayed by the change of OD_600_ per hour using a plate reader. (B) Iron repletion is essential to enable robust yeast growth during ER stress (2 mM DTT) as assayed by the change of OD_600_ per hour using a plate reader. Results are mean±s.d. (*n*=9). **P<0.01 (Student's *t*-test). (C) The presence of iron enables mounting a more effective UPR response as can be seen by measuring the UPRE-GFP reporter over time under various iron repletion or depletion conditions following ER stress (2 mM DTT). (D) Ire1–mCherry clustering following induction of ER stress (2 mM DTT) is affected by the amount of iron in the medium. Scale bar: 5 μm. (E) Iron affects Ire1 clustering in mammalian HeLa cells treated with 10 µg/ml tunicamycin (Tm). While iron depletion (100 µM DFO) disrupts Ire1–GFP clustering, iron repletion (300 µM FAC) enhances it. The bottom row shows a magnification of the Tm-treated cells. Scale bar: 10 μm. (F) Quantification of results in E for DFO=146, FAC=156 and control=135 cells. NT, not treated; WT, wild type.

Since deletion of genes that are important for iron homeostasis may dramatically alter the cellular wiring, we tested whether iron depletion or repletion itself induces the UPR. To this end, we first visualized Ire1–mCherry following iron depletion and found no effect on the localization of Ire1–mCherry in the absence of stress (Fig. S1A). Moreover, we found that neither iron depletion nor repletion had an impact on UPR induction in the asbence of stress as measured by the UPRE-GFP reporter (Fig. S1B). In further support of the notion that iron levels themselves do not stress the ER nor activate the UPR, iron depletion or repletion in the absence of stress also did not affect the growth rate of yeast (Fig. 3A). From these findings, we conclude that iron depletion or repletion does not, it itself, cause ER stress.

**Fig. 4.**
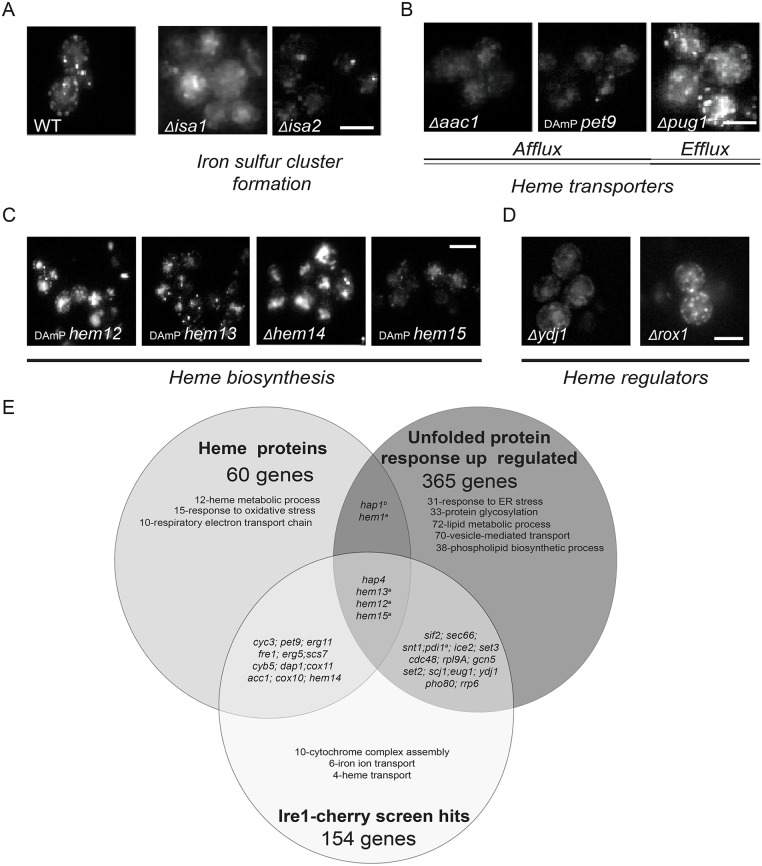
**ISC and heme biosynthesis are essential for Ire1–mCherry clustering.** (A) Deletion of the ISC biosynthesis proteins, *Δisa1* and *Δisa2* abrogates Ire1–mCherry clustering following stress (images from original screen). (B) Reducing heme afflux (Δ*acc1*, *pet9-DAmP*) disrupts Ire1–mCherry clustering following stress, while reducing heme efflux (Δ*pug1*) enhances it (images from original screen). (C) Mutations of the enzymes required for the last steps of the heme biosynthesis pathway disrupt Ire1–mCherry clustering following stress (images from original screen). (D) Deletion of Ydj1, the positive regulator of the heme-specific transcription factor *HAP1*, disrupts Ire1-mCherry clustering following stress, while deletion of the *HAP1* negative regulator, Rox1, enhances it (images from original screen). (E) Venn diagram overlaying heme-containing proteins, UPR upregulated genes and Ire1 clustering effectors from the screen (only the major GO term enrichments are shown for each group). The diagram highlights a potential role for ergosterol biosynthesis (a, hypomorphic allele; b, not in the screen). WT, wild type. Scale bars: 5 μm.

However, upon induction of ER stress, iron-depleted cultures grew poorly, while growth rates improved at high iron concentrations (Fig. 3B). This finding suggests that iron availability is a limiting factor for mounting an effective UPR. Accordingly, while the rate of accumulation of GFP from a UPRE-GFP reporter following stress was similar regardless of iron availability, the levels reached a plateau sooner at high versus low iron concentrations, indicative of a more-timely resolution of the stress (Fig. 4C). Indeed, iron depletion prevented Ire1–mCherry clustering under ER stress conditions, while high iron concentrations promoted Ire1–mCherry clustering with faster kinetics than in standard medium (Fig. 4D; Movies 1 and 2), which could explain why the UPR was more effective in resolving ER stress when iron was abundant.

Most salient features of the *IRE1–HAC1/XBP1* signaling pathway have been conserved from yeast to man, including ER-stress-induced clustering of Ire1/IRE1α (Aragón et al., 2009; Li et al., 2010). To test whether iron levels feed into IRE1α signaling in human cells as well, we employed HeLa cells in which IRE1α was ablated by CRISPR-Cas9 but was reconstituted with murine IRE1α–GFP expressed at near-endogenous levels. We visualized IRE1α–GFP-expressing HeLa cells upon iron depletion [via the iron chelator Deferoxamine (DFO)] or upon iron repletion [via ferric ammonium citrate (FAC)], under basal or ER stress conditions, as elicited by tunicamycin (Tm). Indeed, the dependence of clustering on iron availability was conserved between yeast Ire1 and human IRE1α – iron depletion abolished IRE1α–GFP clustering in response to ER stress, whereas iron repletion led to more robust IRE1α–GFP clustering than in control medium (Fig. 3E,F).

### Heme is a limiting factor for Ire1 clustering

Why is iron affecting Ire1 clustering? Because there is no indication that Ire1 is a metalloprotein, we hypothesized that iron levels may be a limiting factor for Ire1 clustering and activation in an indirect manner. Our GO term enrichment analysis pointed to heme biosynthesis as a central iron-dependent process that affects the UPR (Fig. 2A). In that respect, it was noteworthy that among the hits from our screen were two genes, *ISA1* and *ISA2*, which relate to iron-sulfur cluster (ISC) formation. Deletion of either gene impeded clustering of Ire1–mCherry (Fig. 4A). ISCs serve as co-factors for a wide variety of enzymes, including those responsible for heme biosynthesis. Moreover, several hits from our screen represented ablations of genes encoding heme-containing proteins or enzymes involved in heme biosynthesis. Specifically, we found that mutations in Aac1 and Pet9, the two importers of the heme-precursor into mitochondria (Fig. 4B) caused reduced clustering of Ire1–mCherry. Conversely, deletion of Pug1, a protein that facilitates heme export from the cell, dramatically enhanced Ire1–mCherry clustering (Fig. 4B), which likewise supports the notion that Ire1 clustering propensity correlates with heme availability. Additional hits from our screen further implicated heme homeostasis as a key determinant in the ER stress responsiveness of the Ire1 pathway, since deletions of the genes that encode enzymes that control heme biosynthesis in mitochondria, Hem12, Hem13, Hem14 and Hem15, all caused a reduced clustering of Ire1–mCherry (Fig. 4C).

Regulators of heme biosynthesis also affected Ire1 clustering. Rox1 represses Hem13 transcription when heme levels are elevated (Zhang and Hach, 1999; Zitomer and Lowry, 1992). Deletion of Rox1 thus leads to increased intracellular heme levels, and, accordingly, promoted Ire1–mCherry clustering (Fig. 4D). The heme-activated transcriptional factor Hap1 translocates to the nucleus to drive Rox1 expression, but Ydj1 counteracts Hap1 activity by sequestering it in the cytosol (Lan et al., 2004). Hence, deletion of Ydj1 should lead to increased Rox1 levels and thereby to lowered heme biosynthesis. Indeed, Ire1–mCherry clustering was impeded when Ydj1 was ablated (Fig. 4D). In summary, our findings suggest that heme availability is a limiting factor for the responsiveness of Ire1 to ER stress. Hence, while we do not show a causal and direct connection between iron levels and heme availability, it would be tempting to hypothesize that the requirement for iron in UPR activation is due to its essential role in heme biosynthesis

Why would heme have such an important role in UPR activation? To gain insight into this question, we mapped the overlap between all known heme-containing proteins (Kaniak-Golik and Skoneczna, 2015), UPR targets (Travers et al., 2000) and the hits from our screen. Strikingly, we observed that iron repletion mechanisms are activated by the UPR as is the transcription of many heme biosynthetic enzymes. Hence, there is cross-talk between iron/heme and ER homeostasis: iron/heme levels affect the UPR, and the UPR promotes iron repletion and heme biosynthesis (Fig. 4E). Importantly, we noticed that several hits from our screen were heme-dependent enzymes, among them the ergosterol biosynthetic enzymes, Erg5 and Erg11 (Fig. 4E). These results led us to hypothesize that the dependence on heme could be due to the essential role of heme in sterol biosynthesis.

### Sterol biosynthesis affects Ire1 clustering

**Fig. 5.**
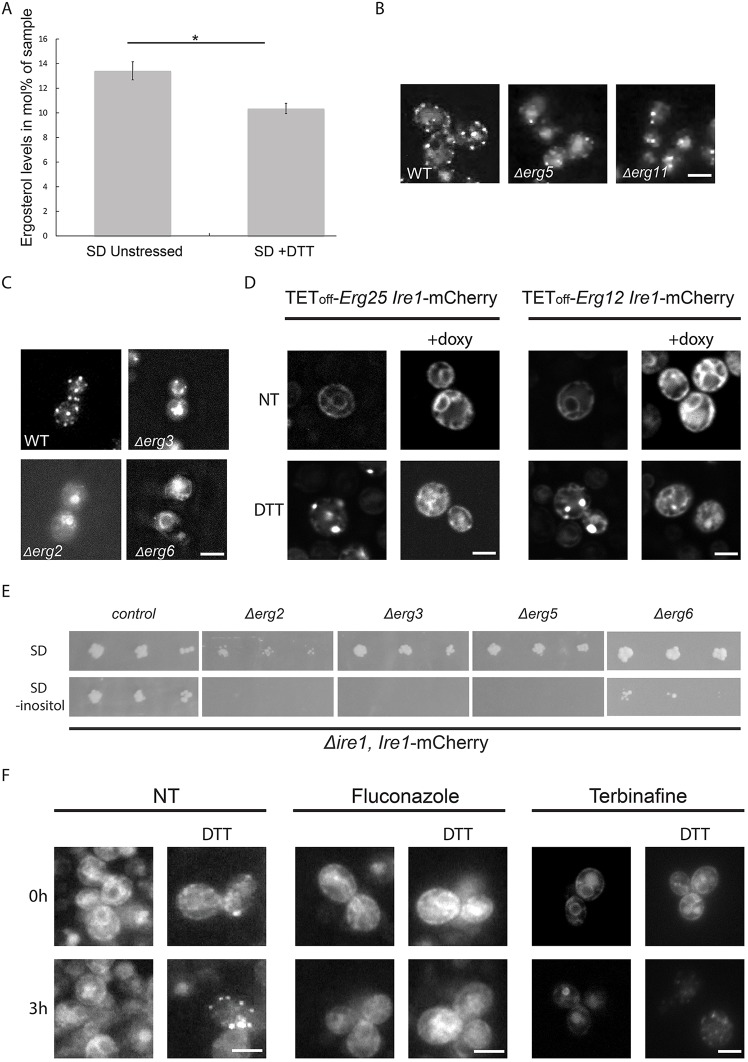
**Ergosterol biosynthesis affects Ire1-mCherry clustering.** (A) ER stress causes a rapid and significant decrease in overall ergosterol levels. Analysis of the ergosterol content is shown in unstressed cells and following 2 mM DTT treatment for 1 h. Results are mean±s.d. (*n*=3). *P<0.05 (Student's *t*-test). (B) Deletions of the heme-containing ergosterol biosynthesis pathway enzymes Erg5 and Erg11 disrupt Ire1–mCherry clustering following ER stress (images from original screen). (C) Deletions of the non-essential ergosterol biosynthesis pathway enzymes, Erg2, Erg3 and Erg6, disrupt Ire1–mCherry clustering following ER stress (2 mM DTT). (D) Repressing the expression of the two essential ergosterol biosynthesis pathway enzymes Erg12 and Erg25 using a TetOFF promotor repressed by doxycycline disrupts Ire1–mCherry clustering following ER stress (2 mM DTT). (E) Deletion strains of the non-essential components of the ergosterol biosynthesis pathway cannot grow even in mild ER stress conditions caused by removal of inositol from the medium (SD –inositol). (F) Short-term inhibition of ergosterol biosynthesis using two different inhibitors (20 μg/ml Fluconazole or 10 μg/ml Terbinafine) disrupts Ire1–mCherry clustering following UPR induction (2 mM DTT). NT, not treated; WT, wild type. Scale bars: 5 μm.

Sterol levels, and more generally, collective membrane properties such as fluidity, have previously been suggested to act as important modulators of ER stress signaling (Pineau et al., 2009; Promlek et al., 2011; Surma et al., 2013; Thibault et al., 2012; Volmer et al., 2013). Indeed, we have recently shown that such bi-layer properties are directly sensed by the unique trans-membrane region of Ire1 (Halbleib et al., 2017). We hence decided to follow sterol levels following induction of ER stress and found that there is a rapid and dramatic decrease in ER membrane sterol levels (Fig. 5A; Table S2). This drop of membrane sterols is remarkable, because previous work by others showed that membrane sterols are kept at a constant level under a wide variety of growth conditions (Klose et al., 2012). This suggests that there is a strong requirement for upregulated sterol biosynthesis to support Ire1–mCherry cluster formation at later times following the stress.

In support of this was the fact that deletions of both heme-containing ergosterol biosynthesis enzymes, Erg5 and Erg11, came up in our screen as preventing Ire1–mCherry clustering (Fig. 5B) similar to the effect of iron or heme depletion. These findings led us to hypothesize that the ergosterol content in the membrane (either directly or indirectly) is an additional factor affecting Ire1 clustering and, thereby, UPR signaling. To support this notion, we first manually deleted three additional non-essential ergosterol biosynthesis enzymes (Fig. 5C) and additionally created ‘shutdown’ alleles for two essential ergosterol biosynthesis enzymes (Fig. 5D). Regardless of the enzyme whose function we perturbed, we consistently ablated Ire1 clustering. Hence, it is not the accumulation or reduction in any specific sterol species but rather a depletion of overall sterol levels or a reduction in biosynthesis rates that is the underlying cause for loss of clustering.

Moreover, loss of any non-essential ergosterol-pathway enzyme led to cells having a complete inability to survive even the mildest of ER stresses, such as that caused by inositol depletion (Cox et al., 1997; Lajoie et al., 2012) (Fig. 5E). This stresses the requirement for optimal *de novo* ergosterol biosynthesis capacities to mount a functional homeostatic response.

However, genetic alterations to the ergosterol pathway cause many pleiotropic effects, making it hard to gauge whether the effects on Ire1 clustering are direct or indirect. To test the role of ergosterol more directly, we employed two ergosterol synthesis inhibitors working on different steps in the pathway: fluconazole (Erg11 inhibitor) and Terbinafine (Erg1 inhibitor). Treatment with either drug confirmed that, under ER stress conditions, Ire1–mCherry no longer clustered when ergosterol biosynthesis was inhibited (Fig. 5F) and the UPR was not efficiently activated (Fig. S2).

### A feedback loop exists between ER stress, iron levels and ergosterol biosynthesis

**Fig. 6.**
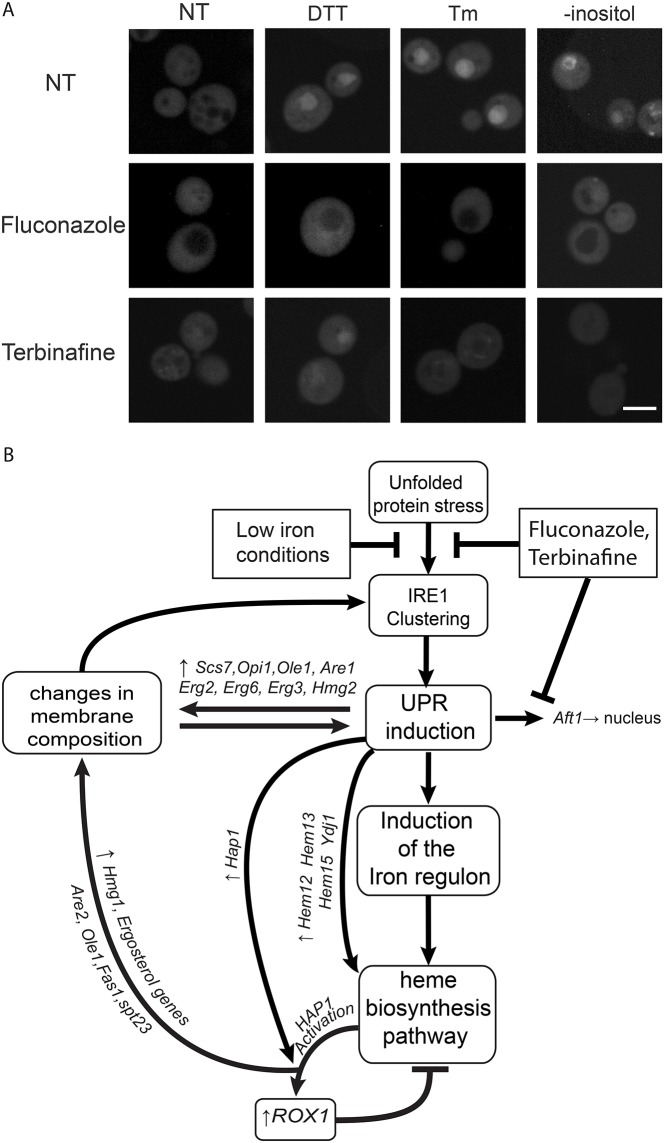
**Suggested cross-talk between the UPR, heme and iron homeostatic machinery.** (A) GFP–Aft1 re-localizes to the nucleus during unfolded protein stress, as induced by either 2 mM DTT, 5 μg/ml tunicamycin or 24 h growth in inositol depletion medium. The nuclear accumulation is inhibited by lowering ergosterol biosynthesis by using ergosterol biosynthesis inhibitors (Fluconazole 20 μg/ml or Terbinafine 10 μg/ml). NT, not treated. Scale bar: 5 μm. (B) A schematic for a hypothetical feedback loop suggested for the heme and iron cross-talk with ergosterol biosynthesis and the UPR.

If iron is essential for mounting a full-blown UPR, then it stands to reason that the cell would regulate iron uptake when the UPR is induced as we have seen (Fig. 4E). Indeed, we found that the major iron regulon inducer, the transcription factor Aft1 (Yamaguchi-Iwai et al., 2002), translocates to the nucleus following ER stress (Fig. 6A). Moreover, some of the targets of Aft1 also came up as hits in the screen suggesting that their upregulation is required to sustain Ire1 clustering (Table S3). Interestingly, ablation of sterol biosynthesis concomitantly with the onset of ER stress suppressed Aft1 nuclear localization suggesting that Aft1 translocation to the nucleus is not a direct result of ER stress sensing but rather a secondary effect of upregulated ergosterol biosynthesis (Fig. 6A).

Taken together, all of our above findings, while not directly showing a causal connection between iron, heme and ergosterol, suggest a testable model whereby unfolded protein stress, iron uptake/utilization, heme biosynthesis and ergosterol build-up are all interconnected through a feedback loop (Fig. 6B).

## DISCUSSION

Our screen unveiled that Ire1 clustering is affected by ablation of a wide range of proteins, among them those that play a role in chromatin remodeling, transcription, translation or mRNA stability. Apparently, a broad network of transcription and translation regulates fine-tuning of the UPR, meriting further investigation.

Another striking observation from our screen is that a reduction of Ire1 clustering seems to be controlled by signaling cascades. Amongst our hits were Npr2, a regulator of the TOR pathway (Laxman et al., 2014), Sap155 a regulator of the Sit4 phosphatase (Luke et al., 1996), Reg1 a regulatory subunit of the Glc7 phosphatase (Tu and Carlson, 1995) and the protein kinase Mck1 (Cherry et al., 2012). It is tempting to speculate that these factors could modulate the Ire1 behavior by altering its phosphorylation status, directly or indirectly. The notion that metabolic input may feed in to the phosphorylation status of Ire1 is not unprecedented: in hepatocytes, glucagon-induced protein kinase A (PKA) phosphorylates IRE1α, which then contributes to gluconeogenic transcription via a still poorly understood mechanism (Mao et al., 2011).

We specifically chose to focus on the strongest link that came up from the screen, which was with iron/heme homeostasis. The model that emerged from our data proposes that Ire1 clustering propensity and, hence, the amplitude of Ire1 signaling, is dependent, at least in part, on sterol levels in the ER membrane. Since it is not clear at present whether this is direct or not, we can only speculate that membrane bilayer packing or membrane reshaping have an effect on Ire1 clustering. This notion is in line with other reports showing that desaturation of fatty acyl chains and ergosterol accumulation, which lead to similar membrane packing alterations, dramatically affect Ire1 activation (Surma et al., 2013; Volmer et al., 2013) and ER stress (Deguil et al., 2011; Feng et al., 2003; Gardner and Walter, 2011; Lajoie et al., 2012; Pineau et al., 2009). The transmembrane domain of Ire1, in fact, is unusual in length and sequence (Promlek et al., 2011) and we have recently shown that it can directly sense and respond to altered lipid content (Halbleib et al., 2017). Our data, while not directly showing causality, enable us to put forward a working hypothesis. We suggest that in the absence of sufficient iron, both iron and heme biosynthesis are reduced, which, in turn, compromises synthesis of ergosterol/cholesterol, causing increased fluidity of the ER membrane and, as such, interfering with Ire1 clustering (Fig. 6B). It remains to be elucidated whether Ire1 clustering goes hand-in-hand with membrane reorganization events and whether Ire1 clusters originate at pre-existing sites where the ER membrane composition is such that it can sustain clustering.

Our findings highlight that iron/heme availability are of such importance for mounting an efficient UPR that they themselves are controlled by UPR activation creating a feedback loop (Fig. 6B). An involvement of heme in modulating the UPR has been reported before: reduced heme levels activate heme-regulated inhibitor (HRI, also known as EIF2AK1) in mammalian cells that in turn causes phosphorylation of eIF2α, triggering activation of the integrated stress response, which is also invoked by the PERK signaling branch of the UPR (Han et al., 2001). Our results now suggest a mechanism for this dependence – through membrane architecture alterations that are dependent on heme-containing enzymes.

The insights that we have gained on the cross-talk between ER stress signaling and iron/heme homeostasis are also interesting in light of human pathogenesis. Enhanced IRE1α clustering and an increased amplitude of UPR signaling caused by iron repletion may well be the basis of the increased risk for patients that suffer from hemochromatosis (i.e. pathological iron overload) of developing type 2 diabetes (Simcox and McClain, 2013; Wang et al., 2015). This condition emerges when ER stress signaling in pancreatic β-cells is excessive, leading to their apoptotic death (Marciniak and Ron, 2006). In further support of how our model may explain the link between excess iron and the onset of diabetes, several lines of evidence indicate that boosting heme oxygenase-1 (HO-1; also known as HMOX1) activity, which lowers heme levels, can alleviate diabetes (Abraham et al., 2008).

More broadly, our findings highlight that stress responses are orchestrated through integration of a variety of homeostatic pathways. They also uncover a role for mitochondria–ER cross-talk in alleviating stress. In the absence of optimal mitochondrial activity, heme biosynthesis will be altered and the ER will not mount the maximal stress response – potentially leaving cellular resources for dealing with mitochondrial stress. Ire1 therefore emerges as a molecular hub that integrates multiple aspects of cell metabolism such that ER stress responses not only meet protein-folding demands but are also in tune with the overall status of the cell.

## MATERIALS AND METHODS

### Yeast strains, strain construction and culturing conditions

All strains in this study are based on the BY4741 laboratory strain (Brachmann et al., 1998). All information on strains, plasmids and primers can be found in Tables S4, S5 and S6.

The libraries used were the yeast deletion library (Giaever et al., 2002) and the DAmP hypomorphic allele library (Breslow et al., 2008). The GFP–Aft1 was taken from the yeast seamless version of the SWAT-GFP library (Yofe et al., 2016).

For the lipidomics assays, a BY4741 WT strain was grown from an optical density at 600 nm (OD_600_) of ∼0.1 to 0.8 in the respective medium and then treated for 1 h with 2 mM DTT.

For the majority of experiments, yeast were grown in standard synthetic dextrose (SD) medium (6.7 g/l yeast nitrogen base with ammonium sulfate, 2% glucose and all necessary amino acids). For inositol-depletion experiments, yeast nitrogen base without inositol (USbiological) was used. Yeast nitrogen base without iron (Sunrise science products) was used for iron depletion and repletion experiments, and supplemented with the indicated concentrations of FeCl_3_ (Sigma). The medium used in the microscopy screening was yeast nitrogen base without riboflavin (Formedium).

DTT (Sigma) was used at 2 mM, tunicamycin (Sigma) was used at 5 µg/ml, Fluconazole (Sigma) was used at 3.3 µg/ml, Terbinafine (Sigma) was used at 10 µg/ml. Repression of the TET promoter for the yeast strains was performed by using 15 µg/ml doxycycline for 11 h.

### Yeast library preparation

To create the collection of haploid strains containing Ire1–mCherry on the background of all yeast mutants, an Ire1–mCherry-expressing query strain was constructed on the basis of a synthetic genetic array (SGA) compatible strain, YMS721 (Papić et al., 2013). Using the SGA method (Cohen and Schuldiner, 2011; Tong and Boone, 2006), the Ire1–mCherry query strain was crossed with the libraries. To perform the SGA in high-density format we used a RoToR bench-top colony arrayer (Singer Instruments). In short, mating was performed on rich medium plates, and selection for diploid cells was performed on SD-URA plates containing geneticin (200 µg/ml). Sporulation was induced by transferring cells to nitrogen starvation medium plates for 7 days. Haploid cells containing the desired mutations were selected by transferring cells to SD-URA plates containing geneticin (200 µg/ml) alongside the toxic amino acid derivatives canavanine and thialysine (Sigma-Aldrich) to select against remaining diploids, and lacking histidine to select for spores with an **a** mating type.

### Automated high-throughput fluorescence microscopy

The collection was visualized using an automated microscopy setup as described previously (Breker et al., 2013). In short, cells were transferred from agar plates into 384-well polystyrene plates for growth in liquid medium using the RoToR arrayer robot. Liquid cultures were grown in a LiCONiC incubator, overnight at 30°C in SD medium lacking uracil to select for yeast containing the plasmid encoding Ire1–mCherry. A JANUS liquid handler (PerkinElmer) connected to the incubator was used to dilute the strains to an OD_600_ of ∼0.2 in plates containing SD plus DTT. Plates were incubated at 30°C for 2 h. The cultures in the plates were then transferred by the liquid handler into glass-bottom 384-well microscope plates (Matrical Bioscience) coated with concanavalin A (Sigma-Aldrich). After 20 min, wells were washed twice with SD −riboflavin complete medium to remove non-adherent cells and to obtain a cell monolayer. The plates were then transferred to the ScanR automated inverted fluorescent microscope system (Olympus) using a robotic swap arm (Hamilton). Images of cells in the 384-well plates were recorded in SD −riboflavin complete medium at 24°C using a 60× air lens (NA 0.9) and with an ORCA-ER charge-coupled device camera (Hamamatsu). Images were acquired in the mCherry channel (excitation filter 572/35 nm, emission filter 632/60 nm).

### Inositol depletion spot assay

For the inositol depletion assays, strains were grown overnight at 30°C in SD medium with the selection required for each strain. The cultures were diluted to an OD_600_ of ∼0.4, 0.2, 0.1 and 0.05. 2 µl of each dilution were placed on an SD plate and an SD −inositol plate. The plates were incubated for 24 h at 30°C and imaged.

### Microscopy of yeast strains

For manual microscopy of yeast strains we used the VisiScope Confocal Cell Explorer system, composed of a Zeiss Yokogawa spinning disk scanning unit (CSU-W1) coupled with an inverted Olympus microscope (IX83; ×60 oil objective; excitation wavelength of 488 nm for GFP and 560 nm for mCherry). Images were taken by a connected PCO-Edge sCMOS camera controlled by VisView software. All the images were processed using Fiji software (Schindelin et al., 2012), the contrast was corrected using the auto adjust function, the background was removed by using a 50 pixel rolling ball. Poisson noise was removed by using the PureDenoise Fiji add-on.

### Ergosterol extraction for mass spectrometry

Mass spectrometry-based ergosterol analysis was performed by Lipotype GmbH (Dresden, Germany) as described previously (Ejsing et al., 2009; Klose et al., 2012). Ergosterols were extracted using a two-step chloroform/methanol procedure (Ejsing et al., 2009). Samples were spiked with the internal standard stigmastatrienol. After extraction, the organic phase was transferred to an infusion plate and dried in a speed vacuum concentrator. In the first step, dry extract was re-suspended in 7.5 mM ammonium acetate in chloroform/methanol/propanol (1:2:4, v/v/v) and in the second step, dry extract in 33% ethanol solution of methylamine in chloroform/methanol (0.003:5:1; v/v/v). All liquid-handling steps were performed using Hamilton Robotics STARlet robotic platform with the Anti Droplet Control feature for organic solvent pipetting.

### MS data acquisition, analysis and post-processing of ergosterol levels

Samples were analyzed by direct infusion on a QExactive mass spectrometer (Thermo Scientific) equipped with a TriVersa NanoMate ion source (Advion Biosciences). Samples were analyzed in both positive and negative ion modes with a resolution power of 280,000 at *m*/*z*=200 for mass spectrometry (MS). MS only was used to monitor ergosterol as a protonated ion of an acetylated derivative (Liebisch et al., 2006).

Data were analyzed with in-house-developed lipid identification software based on LipidXplorer (Herzog et al., 2011, 2012). Data post-processing and normalization were performed using an in-house developed data management system. Only lipid identifications with a signal-to-noise ratio >5, and a signal intensity 5-fold higher than in corresponding blank samples were considered for further data analysis.

### Mammalian cell culture and growth conditions

HeLa cells were genotyped by PCR single locus technology to be HeLa S3. Ire1-KO cells were negatively tested for mycoplasma contamination on Nov 10, 2016 by PCR, on conditioned medium from a weekend culture, using the following primers: myc Fw, 5′-ACTCCTACGGGAGGCAGCAGT-3′; myc Rv, 5′-TGCACCATCTGTCACTCTGTTAACCTC-3′.

Cells were grown in Dulbecco's modified essential medium (DMEM) GlutaMAX (Gibco Life Technologies) supplemented with 5% Tet-System-approved fetal bovine serum (ClonTech), 10 nM doxycycline, 100 U/ml penicillin and 100 μg/ml streptomycin (Pen-Strep, Lonza BioWhittaker). Cells were cultured at 37°C in a humidified 5% CO_2_ incubator and passaged twice a week. Cells between 80% and 100% confluence were washed with PBS without Ca^2+^ and Mg^2+^ (DPBS) to get rid of both medium and dead cells and detached using Trypsin-EDTA (Gibco Life Technologies). After detachment cells were centrifuged (250 ***g*** for 5 min), re-suspended in medium and plated on a new plate. All cell lines used in this study were derived from HeLa cells that were modified by lentiviral infection to express the transgene of interest under inducible promoters. Production of lentiviral vectors was performed as previously described (Dull et al., 1998).

Iron depletion was performed by addition of deferoxamine (DFO; Biofutura Pharma, Milan, Italy). Ferric ammonium citrate (FAC; Sigma-Aldrich) was used for iron-loading experiments. Cells were incubated with 100 µM freshly prepared DFO or 300 µM of FAC overnight and then treated with 10 µg/ml tunicamycin for 4 h.

### Generation of IRE1-KO and reconstituted TetON-mIRE1α-GFP HeLa cells

Using CRISPR/Cas9 methodology, we knocked out IRE1α in HeLa cells, resulting in an IRE1α-knockout (KO) clonal cell line. The CRISPR strategy involved the creation of an indent and a subsequent frameshift at the level of the first exon (target sequence, TCTTGCTTCCAAGCGTATACAGG). Knockout clones were verified by western blotting and by functional assays (i.e. absence of XBP1 splicing upon ER stress). By performing lentiviral transduction, we reconstituted IRE1α-KO-TetON cells by delivery of a cassette of GFP-tagged murine IRE1α (IRE1α–GFP) under control of a ‘tight’ Tet-responsive element (Clontech), yielding clonal TetON-IRE1α-GFP cells. The GFP tag was introduced into the juxtamembrane cytosolic linker domain of IRE1α, where such tagging had been shown before to not interfere with function of human IRE1α * *(Li et al., 2010) or yeast Ire1 (Aragón et al., 2009). As expected, murine IRE1α–GFP clustered upon Tm treatment, similar to human IRE1α–GFP and yeast Ire1–GFP.

### Microscopy of mammalian cell cultures

HeLa cells were grown on coverslips in 12-well plates and fixed in 4% paraformaldehyde for 15 min at room temperature and washed in PBS. Nuclei were stained with DAPI diluted in PBS for 10 min at room temperature in the dark. After washing in PBS, coverslips were mounted on microscope glass slides with Mowiol. Light microscopy images were acquired at the UltraView spinning disk confocal microscope operated by Volocity software (PerkinElmer). A 63× objective was used. GFP was excited with the 488 nm laser line.

### Image analysis

To segment and calculate the area of the puncta in the mammalian cells we used ilastik software (Sommer et al., 2011).

### Calculating induction of the UPR using the UPRE-GFP reporter

Fold UPR activation was calculated in the following way:

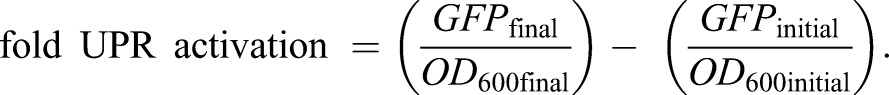


### Enrichment analysis

Enrichment analysis for screen hits was performed by using the GOrilla analysis algorithm (Eden et al., 2007, 2009).

## Supplementary Material

Supplementary information
