# Supplementary material for: Iron affects Ire1 clustering propensity and the amplitude of endoplasmic reticulum stress signaling
Source: J Cell Sci. 2017 Oct 1;130(19):3222–33. doi: 10.1242/jcs.201715 (PMC5665437; doi:10.1242/jcs.201715)
Supplement: Supplementary information [file joces-130-201715-s1.pdf]

Cohen *et al*, Figure S1

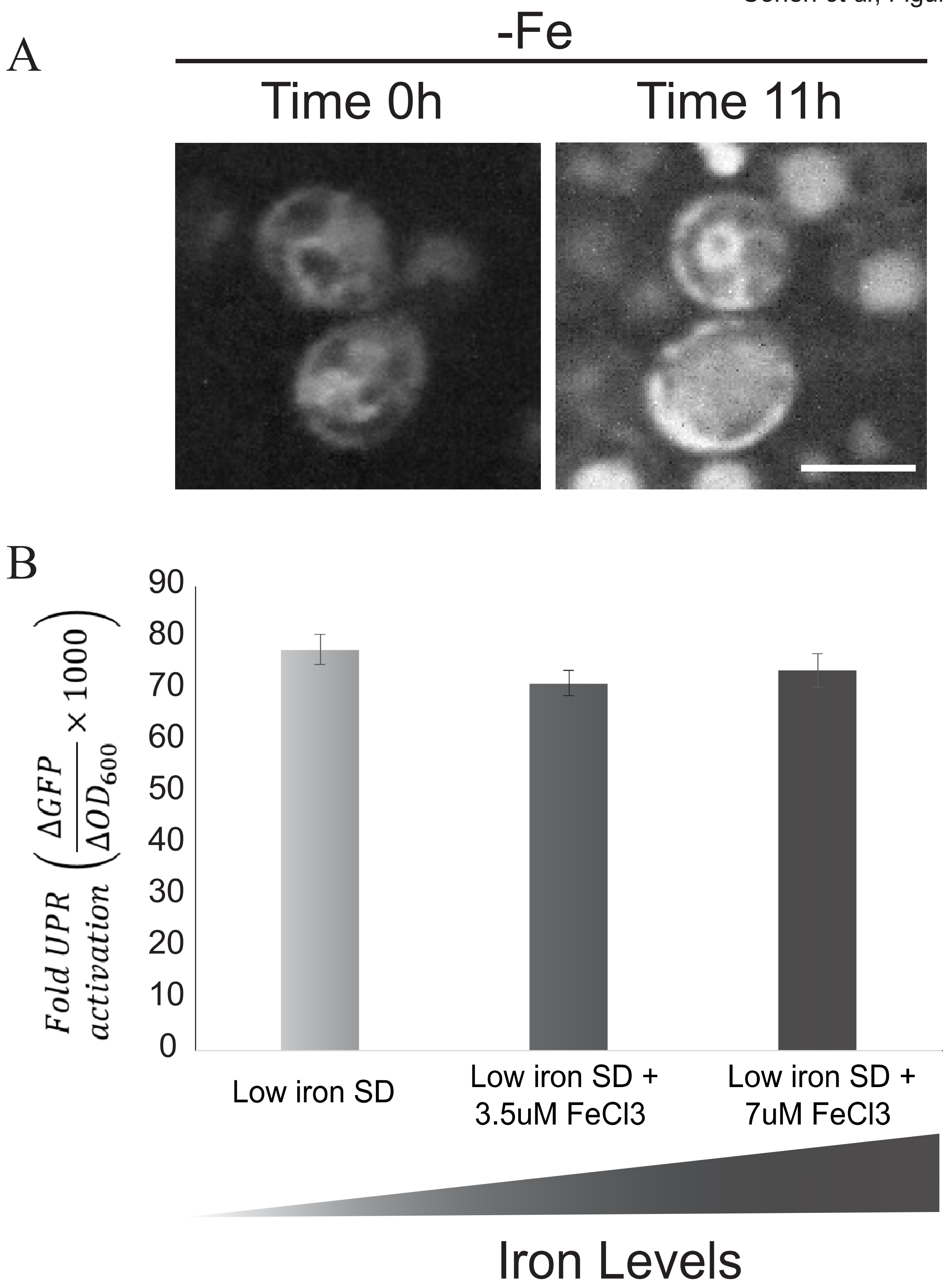

Supplementary Figure 1: Iron does not activate the UPR on its own.

A) Ire1-mCherry visualization during iron depletion shows no change

B) Ire1-mCherry clustering is not affected by the amount of iron in the medium without UPR induction.

Journal of Cell Science • Supplementary information

Cohen *et al*, Figure S2

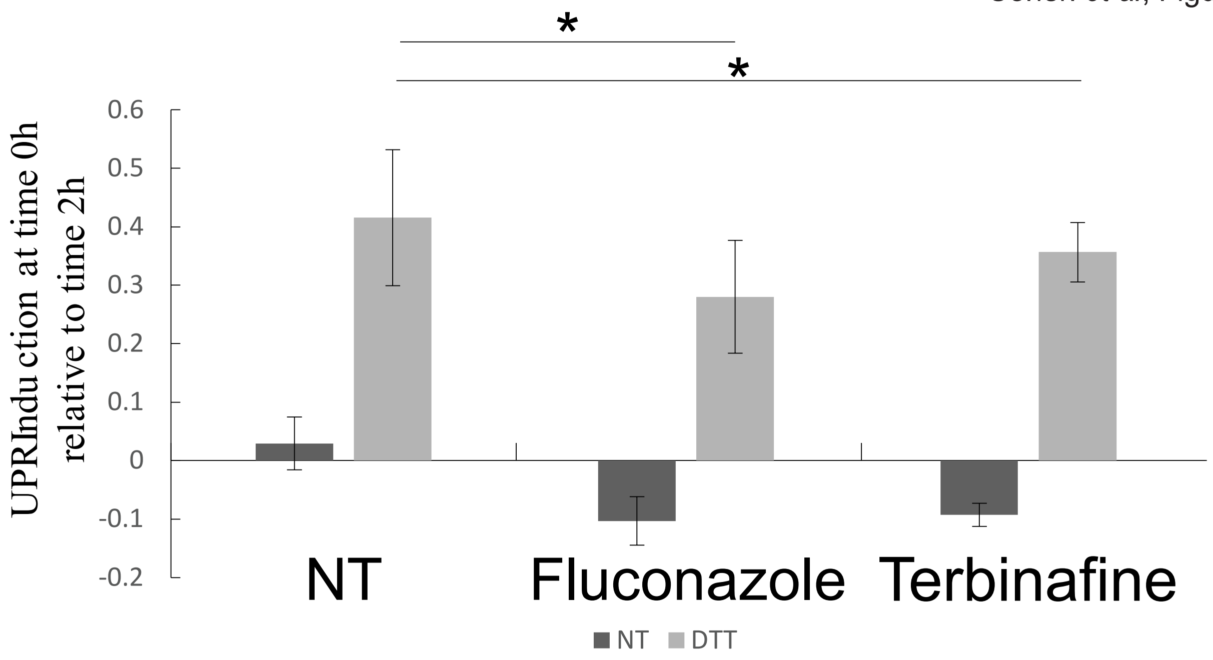

Supplementary Figure 2: Ergosterol inhibitors reduce UPR activation.
The ergosterol inhibitors, fluconazole and terbinafine, reduce the effective UPR response as can be seen by measuring a UPRE-GFP reporter normalized to cytosolic mCherry over time at unstressed conditions and following ER stress (2mM DTT).

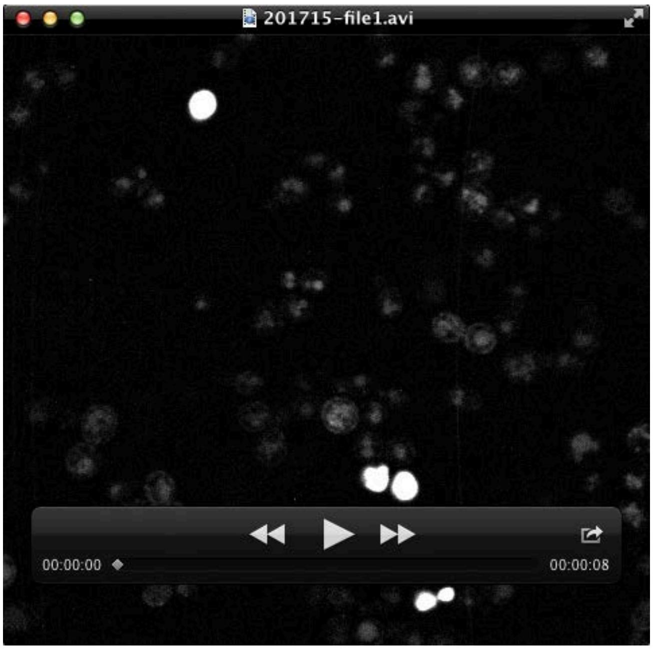

Supplementary Movie 1: Time lapse of Ire1-mCherry clustering following 2mM DTT treatment over 14 hours.

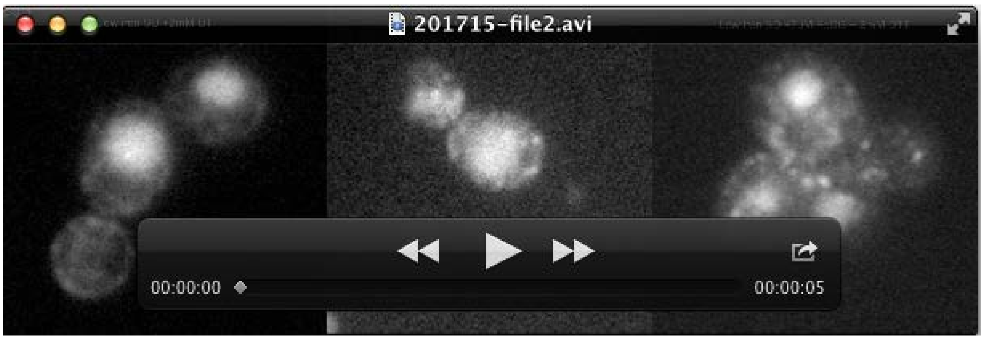

Supplementary Movie 2: Time lapse of Ire1-mCherry clustering following 2mM DTT treatment over two hours in iron repletion (Low iron SD + 7µm FeCl3 +2mM DTT) or iron depletion conditions (Low iron SD +2mM DTT) and their control (Low iron SD + 3.5µm FeCl3 +2mM DTT) (iron concentrations identical to regular SD).

Table S1-S6

Click here to Download Tables S1 - S6

Journal of Cell Science • Supplementary information
